# Factors associated with vitamin D levels in Mongolian patients with multiple sclerosis

**DOI:** 10.1371/journal.pone.0317279

**Published:** 2025-01-24

**Authors:** Myadagmaa Jaalkhorol, Amarsaikhan Dashtseren, Gantuya Magnaibayar, Badrangui Bat-Orgil, Ikuo Tsunoda, Shiirevnyamba Avirmed, Stefania Iaquinto, Viktor von Wyl

**Affiliations:** 1 Department of Health Research, Graduate School, Mongolian National University of Medical Sciences, Ulaanbaatar, Mongolia; 2 Mongolian Naran Multiple Sclerosis Society, Ulaanbaatar, Mongolia; 3 Department of Preventive Medicine, School of Public Health, Mongolian National University of Medical Sciences, Ulaanbaatar, Mongolia; 4 Center of Neurology, General Hospital for State Special Servants, Ulaanbaatar, Mongolia; 5 Department of Life Sciences, Human International School, Ulaanbaatar, Mongolia; 6 Department of Microbiology, Kindai University Faculty of Medicine, Osakasayama, Osaka, Japan; 7 Graduate School, Mongolian National University of Medical Sciences, Ulaanbaatar, Mongolia; 8 Department of Epidemiology, Epidemiology Biostatistics and Prevention Institute, University of Zurich, Zurich, Switzerland; 9 Institute for Implementation Science in Health Care, University of Zurich, Zurich, Switzerland; West Bengal University of Animal and Fishery Sciences, INDIA

## Abstract

**Background:**

Multiple sclerosis (MS) onset is caused by genetic and environmental factors. Vitamin D has been identified as contributing environmental risk factor, with higher prevalence at latitudes further from the equator. Mongolia, at 45°N, has limited sunlight exposure, increasing the population’s risk for vitamin D deficiency.

**Objectives:**

To compare vitamin D levels between persons with MS (pwMS) and persons without MS and to identify factors associated with low vitamin D.

**Methods:**

We investigated associations with vitamin D levels using data from MS cases and controls from Mongolia. We used linear mixed-effects regression with fixed effects (case status, sociodemographics, and predefined variables) and participant-specific random intercepts.

**Results:**

Of 62 participants (31 pwMS, 31 controls), pwMS had lower summer [median 23.00 ng/ml (interquartile range 11.30–31.50) vs. 25.00 ng/ml (19.25–32.00)] and winter vitamin D levels [21.00 ng/ml (10.60–27.60) vs. 23.50 ng/ml (15.55–28.60)], with a smaller seasonal decline. Vitamin D deficiency was more prevalent in pwMS. None of these findings reached statistical significance. Winter season and being breastfed as a child were associated with significantly lower vitamin D levels.

**Conclusion:**

Vitamin D deficiency was common among pwMS, which could be influenced by behavioural factors. These findings may inform more targeted recommendations for pwMS to maintain sufficient vitamin D levels.

## Introduction

Multiple sclerosis (MS) is a chronic inflammatory demyelinating disease of the central nervous system. It affects twice as many women as men of working age between 20 and 40 years and is a major cause of work disability among young people [1].

Although the precise cause of MS is still unknown, genetic and environmental factors, such as smoking [2, 3] or Epstein-Barr virus infection [4], are believed to influence the development of the disease [5, 6]. Furthermore, regions with less sunlight have a higher incidence of vitamin D deficiency, as well as MS [7]. Vitamin D is involved in the metabolism of calcium and phosphorus. Its deficiency is manifested by weakness in children and osteoporosis in adults [8]. Vitamin D has also been demonstrated to regulate the development and reproduction of immune cells. Therefore, a lack of vitamin D could lead to the development of autoimmune diseases. The active form of vitamin D can modulate T helper (Th) 1 cells, which can play a key role in the pathogenesis of MS [9, 10]. In the human body, 80–90% of vitamin D is synthesized in the skin through exposure to ultraviolet rays. The remaining 10–20% is obtained from food [11].

Several studies suggest an impact of vitamin D on the development of MS and disease progression. For example, Fahmi et al. and Kutlu et al. showed that vitamin D levels were significantly decreased in MS patients and were correlated with MS severity [12, 13]. In addition, Smolders et al. reported an association of serum vitamin D levels with the number of seizures and disability in patients with MS [8].

In people located at latitudes above 37° N, the skin synthesis of vitamin D is generally reduced due to a weaker sun ultraviolet reflection [14]. Because Mongolia is located at a latitude of 45° N, its inhabitants are at higher risk of vitamin D deficiency. Furthermore, the consumption of vitamin D-rich foods such as fish and eggs is generally low in Mongolia [15]. However, the epidemiology of MS in Mongolia, as well as the distribution and role of key risk factors, including vitamin D levels, remain unclear [16]. Therefore, using a case-control design, we aimed to compare vitamin D levels between people with MS (cases) and people without MS (controls), and to identify factors associated with vitamin D deficiency.

## Materials and methods

### Study design, setting, participants, and questionnaire

We conducted a case-control study (medical records based on control materials and identified each recorded case report) at the Health Units of Ulaanbaatar, Mongolia. We recruited 31 MS patients (26 females and five males) from six Health Units in Ulaanbaatar (Khan-Uul, Songinokhairkhan, Sukhbaatar, Bayangol, Bayanzurkh, and Chingeltei) for the case group. Of these, 20 were diagnosed with relapsing-remitting (RR)-MS, and 11 with secondary progressive (SP)-MS. We used McDonald criteria [17] to diagnose MS patients who resided in the Ulaanbaatar district, attended the district hospital in Ulaanbaatar and were at least 18 years old. We excluded patients who had refused to participate in the study or were under 18 years old. Additionally, we excluded patients who did not belong to the Ulaanbaatar district to avoid the potential influence of varying geographical and environmental factors, including altitude and temperature, across the local districts of Mongolia. The controls (23 females and eight males) were recruited among patients without MS who were seeking care at the same hospital on January 20, 2023, and matched to cases by sex and age. The authors responsible for data collection had access to information that could identify individual participants during and after data collection.

Cases and controls were selected by retrospectively searching medical records based on the study inclusion criteria. The eligible cases and controls were directly contacted by telephone and invited to an on-site enrolment visit, at which the informed consent was signed. All persons who were contacted agreed to participate in the study and to provide two blood samples at different time points. Therefore, there were no exclusions or dropouts during our study. Of note, we had initially specified in the study protocol that participants taking vitamin D and calcium supplements should be excluded. However, we later realized that 6 cases and 5 controls reported taking vitamin D in the surveys. Given our limited sample size and since the number of cases and controls taking vitamin D were balanced, we decided to keep these individuals in our study.

Data for MS patients and controls was collected by trained nurses at the hospital through detailed interviews using a structured questionnaire. All participants completed a questionnaire covering information on their demographics and lifestyle (education, marital status, employment, living situation, smoking, alcohol, religion, and ethnicity). In addition, MS cases provided clinical information (disease onset and duration, family history of MS, treatment, comorbidity, neurological signs including deep tendon reflex, vision, pyramidal and brainstem sign/symptom, sensation, disequilibrium, and urination). The questionnaire was conducted within 20–30 minutes from the 20th of January 2023 to the 20th of July 2023. All participants provided written informed consent before participating in the study.

The study protocol was approved by the Ethics Committee of the Mongolian National University of Medical Sciences (MNUMS, No.: 2023/01/20–2023/D-03).

### EDSS examinations

The Expanded Disability Status Scale (EDSS) score [18] was assessed by trained neurologists to determine the disability levels of the MS patients in eight functional systems (pyramidal, cerebellar, brainstem, sensory, bowel and bladder, visual, cerebral, and other) and assigned a score to each functional system. The EDSS score ranges from 0 (normal) to 10 (death due to MS), with half-step score increments. We classified the MS patients into two groups based on the disability levels assessed by the EDSS: “mild disability” for a score < 5 and “moderate to severe disability” for a score ≥ 5.

### Outcome measures

The primary outcome of the present study was serum vitamin D level, which was measured repeatedly at two distinct visits in winter and in summer to capture seasonal variability. Therefore, two measurements of serum vitamin D levels were obtained for each participant. Blood samples were coded for each person and immediately transported to the laboratory of the Nomun Hospital, where serum vitamin D (25-hydroxyvitamin D) levels were measured by enzyme-linked fluorescence analysis (ELFA) in the fully automated immunological analyzer Vidas (Biomerieux, Marcy-L’Etoile, France). Serum vitamin D levels were categorized as “deficient” (< 20 ng/ml), “insufficient” (21–30 ng/ml), or “sufficient” (> 30 ng/ml), according to the guidelines of the International Osteoporosis Foundation (IOF) and the European Society of Endocrinology (ESE) [19, 20].

### Variables of interest

The main variable of interest was group status, which differentiated individuals with MS from individuals without MS. We further explored associations of participants’ characteristics with serum vitamin D levels by examining a comprehensive set of variables, organized into three main categories: 1) sociodemographic factors [age, sex (female or male), highest education level (basic education, high school, or bachelor and higher), employment status (employed/self-employed, retired, or disabled), income, and marital status (single/divorced/widowed or married/cohabitant)]; 2) health-related factors [body mass index (BMI) and blood group (A, B, AB, or O)]; and 3) lifestyle factor [dietary habits (frequency of fish, egg, or milk consumption), intake of vitamin D supplements (taking vitamin D supplements or not), daily sun exposure (more or less than 30 minutes), season of birth (winter, spring, summer or autumn), smoking status (current smoker or non-smoker), and alcohol consumption (currently drinking alcohol or not)].

### Statistical analysis

Descriptive statistics were used to compare sociodemographic and health-related characteristics between persons with MS and persons without MS. Categorical variables were summarized as numbers and percentages, and continuous variables were summarized as median and interquartile ranges or mean and standard deviations. Univariate tests were performed to assess differences in the measured variables between persons with MS and persons without MS. The Mann-Whitney U test was used for continuous variables, and Fisher’s exact test was used for categorical variables.

We used linear mixed-effects regression to analyse associations between the variables of interest and serum vitamin D levels. Pre-defined fixed effects included the group indicator (MS cases vs. controls), sex (female vs. male), age, and smoking status (current smoker vs. non-smoker), with a participant-specific random intercept to account for within-subject variability, given that serum vitamin D level was measured repeatedly for each participant during winter and summer. Additional variables were added sequentially in a bottom-up variable selection approach and retained in the model if the Akaike Information Criterion (AIC) decreased by two or more units. The final linear mixed-effects model included the following fixed effects: season, group, age, gender, smoking status, vitamin D supplementation, marital status, and breastfeeding history. In addition, an interaction effect between season and group was included in the model.

As a sensitivity analysis, we additionally conducted a generalized linear mixed-effects regression, where serum vitamin D levels were categorized into a binary outcome (non-deficient: > 30ng/ml vs. deficient ≤ 30 ng/ml)). Furthermore, we conducted another sensitivity analysis, excluding individuals who reported taking vitamin D supplements.

Post-hoc power analyses were conducted to assess the adequacy of the sample size for detecting the effects of interest. Specifically, we calculated the required sample size to achieve 80% power for both the main effect of disease status (MS vs. control) and the interaction effect between season and disease status.

All statistical analyses were performed using R (Version 4.2.2) and R Studio (Version 2022.12.0+353) software.

## Results

### Study population

The characteristics of the study population are displayed in Table 1. Our study included 62 participants, of which 31 individuals were diagnosed with MS (cases), and the other 31 individuals were without the disease (controls). The MS cases were slightly older than the control group [median 50 (interquartile range) (38.5–55.5) vs. 47 (37.0–55.0), *p* = 0.773] with a greater proportion of women (83.9% vs. 74.2%, *p* = 0.533). A notable difference between persons with MS and persons without MS was observed in the employment status. The majority of persons with MS were either disabled (61.3%) or retired (16.1%), while all controls were employed (*p* < 0.001). This difference also extends to income levels, which were lower in the MS group (*p* < 0.001). Lifestyle behaviors, including smoking (*p* = 0.605), were similar between the groups. However, there was a marked difference in alcohol intake (*p* = 0.009) and sun exposure. Only 22.6% of the participants with MS reported more than 30 minutes of daily sun exposure, whereas all individuals in the control group reported more than 30 minutes of sun exposure per day (100%) (*p* < 0.001).

**Table 1:** Comparison of persons with MS (cases) and persons without MS (controls).

	Case	Control	*p*-value
N	31	31	
Sociodemographic characteristics			
Female, N (%)	26 (83.9)	23 (74.2)	0.533
Age, median [IQR]	50 [38.5, 55.5]	47 [37.0, 55.0]	0.773
Education level, N (%)			0.017
Basic education	12 (38.7)	19 (61.3)	
High school	19 (61.3)	9 (29.0)	
Bachelor or higher	0 (0.0)	3 (9.7)	
Employment status, N (%)			<0.001
Employed	7 (22.6)	31 (100)	
Retired	5 (16.1)	0 (0.0)	
Disabled	19 (61.3)	0 (0.0)	
Monthly income in USD, N (%)			<0.001
< 161.196	19 (61.3)	2 (6.5)	
161.196–307.737	7 (22.6)	13 (41.9)	
307.737–454.279	5 (16.1)	13 (41.9)	
> 454.279	0 (0.0)	3 (9.7)	
Living situation, N (%)			<0.001
Ger house	4 (12.9)	16 (51.6)	
House	7 (22.6)	10 (32.3)	
Apartment	20 (64.5)	5 (16.1)	
Marital status, N (%)			0.198
Single / divorced / widowed	10 (32.3)	16 (51.6)	
Married / cohabitant	21 (67.7)	15 (48.4)	
Season of birth, N (%)			0.611
Spring	3 (9.7)	6 (19.4)	
Summer	5 (16.1)	3 (9.7)	
Autumn	8 (25.8)	6 (19.4)	
Winter	15 (48.4)	16 (51.6)	
Being breastfed as a child, N (%)	26 (83.9)	28 (90.3)	0.705
Comorbidities			
Comorbidities, N (%)			0.091
Cardiovascular disease	5 (16.1)	9 (29.0)	
Cancer	1 (3.2)	0 (0.0)	
Diabetes mellitus	1 (3.2)	6 (19.4)	
Autoimmune diseases	9 (29.0)	6 (19.4)	
Ophthalmic disease	7 (22.6)	7 (22.6)	
Allergy	8 (25.8)	2 (6.5)	
Other	0 (0.0)	1 (3.2)	
Lifestyle factors			
Currently smoking, N (%)	11 (35.5)	14 (45.2)	0.605
Alcohol, N (%)	3 (9.7)	13 (41.9)	0.009
Sun exposure > 30 minutes per day, N (%)	7 (22.6)	31 (100)	<0.001
Traveling to countryside in summer, N (%)	22 (71.0)	24 (77.4)	0.772
Nutrition			
Taking vitamin D supplements, N (%)	6 (19.4)	5 (16.1)	1.000
Beef consumption, N (%)			0.001
Never	1 (3.2)	2 (6.5)	
Occasionally (1-4/month)	4 (12.9)	11 (35.5)	
Regularly (2-6/week)	8 (25.8)	15 (48.4)	
Frequently (>6/week)	18 (58.1)	3 (9.7)	
Mutton consumption, N (%)			<0.001
Never	4 (12.9)	1 (3.2)	
Occasionally (1-4/month)	4 (12.9)	7 (22.6)	
Regularly (2-6/week)	2 (6.5)	18 (58.1)	
Frequently (>6/week)	21 (67.7)	5 (16.1)	
Goat consumption, N (%)			0.048
Never	21 (67.7)	16 (51.6)	
Occasionally (1-4/month)	10 (32.3)	8 (25.8)	
Regularly (2-6/week)	0 (0.0)	6 (19.4)	
Frequently (>6/week)	0 (0.0)	1 (3.2)	
Pork consumption, N (%)			0.192
Never	16 (51.6)	22 (71.0)	
Occasionally (1-4/month)	15 (48.4)	9 (29.0)	
Regularly (2-6/week)	0 (0.0)	0 (0.0)	
Frequently (>6/week)	0 (0.0)	0 (0.0)	
Chicken consumption, N (%)			<0.001
Never	3 (9.7)	18 (58.1)	
Occasionally (1-4/month)	23 (74.2)	12 (38.7)	
Regularly (2-6/week)	5 (16.1)	1 (3.2)	
Frequently (>6/week)	0 (0.0)	0 (0.0)	
Fish consumption, N (%)			0.068
Never	10 (32.3)	2 (6.5)	
Occasionally (1-4/month)	14 (45.2)	18 (58.1)	
Regularly (2-6/week)	6 (19.4)	8 (25.8)	
Frequently (>6/week)	1 (3.2)	3 (9.7)	
Egg consumption, N (%)			0.041
Never	3 (9.7)	2 (6.5)	
Occasionally (1-4/month)	7 (22.6)	18 (58.1)	
Regularly (2-6/week)	14 (45.2)	8 (25.8)	
Frequently (>6/week)	7 (22.6)	3 (9.7)	
Milk consumption, N (%)			0.043
Never	13 (41.9)	18 (58.1)	
Occasionally (1-4/month)	18 (58.1)	10 (32.3)	
Regularly (2-6/week)	0 (0.0)	3 (9.7)	
Frequently (>6/week)	0 (0.0)	0 (0.0)	
Cheese consumption, N (%)			0.799
Never	15 (48.4)	17 (54.8)	
Occasionally (1-4/month)	16 (51.6)	14 (45.2)	
Regularly (2-6/week)	0 (0.0)	0 (0.0)	
Frequently (>6/week)	0 (0.0)	0 (0.0)	
Medical parameters			
Serum vitamin D (ng/ml) in summer, median [IQR]	23.00 [11.30, 31.50]	25.00 [19.25, 32.00]	0.234
Serum vitamin D (ng/ml) in winter, median [IQR]	21.00 [10.60, 27.60]	23.50 [15.55, 28.60]	0.301
Serum vitamin D summer-winter difference, mean (SD)	1.92 (2.45)	3.12 (3.28)	0.109
Vitamin D levels summer, N (%)			0.631
Deficient (< 20 ng/ml)	13 (41.9)	10 (32.3)	
Insufficient (21–30 ng/ml)	7 (22.6)	10 (32.3)	
Sufficient (> 30 ng/ml)	11 (35.5)	11 (35.5)	
Vitamin D levels winter, N (%)			0.696
Deficient (< 20 ng/ml)	15 (48.4)	12 (38.7)	
Insufficient (21–30 ng/ml)	10 (32.3)	13 (41.9)	
Sufficient (> 30 ng/ml)	6 (19.4)	6 (19.4)	
Height, median [IQR]	160.00 [156.50, 166.00]	163.00 [158.50, 168.00]	0.290
Weight, median [IQR]	63.00 [57.50, 71.50]	73.00 [68.50, 80.00]	0.002
Body Mass Index, median [IQR]	24.61 [22.23, 27.25]	29.05 [25.63, 29.88]	0.002
Blood group, N (%)			0.620
O	8 (25.8)	9 (29.0)	
A	9 (29.0)	7 (22.6)	
B	12 (38.7)	10 (32.3)	
AB	2 (6.5)	5 (16.1)	
Ability to walk, N (%)	23 (74.2)	31 (100)	0.008
Using a wheelchair, N (%)	8 (25.8)	0 (0.0)	0.008
MS-related factors			
Age at MS onset, median [IQR]	35 [28.5, 38.5]	NA	NA
MS type, N (%)			
Relapsing-remitting MS	20 (64.5)	NA	NA
Secondary-progressive MS	11 (35.5)	NA	NA
Expanded Disability Status Scale (EDSS), median [IQR]	4.5 [2.75, 6.25]	NA	NA

IQR: Interquartile range, NA: not applicable. *p*-values were calculated using Fisher’s exact test for categorical variables and the Mann-Whitney U test for continuous variables.

### Differences in vitamin D levels between MS and control groups

**Fig 1:**
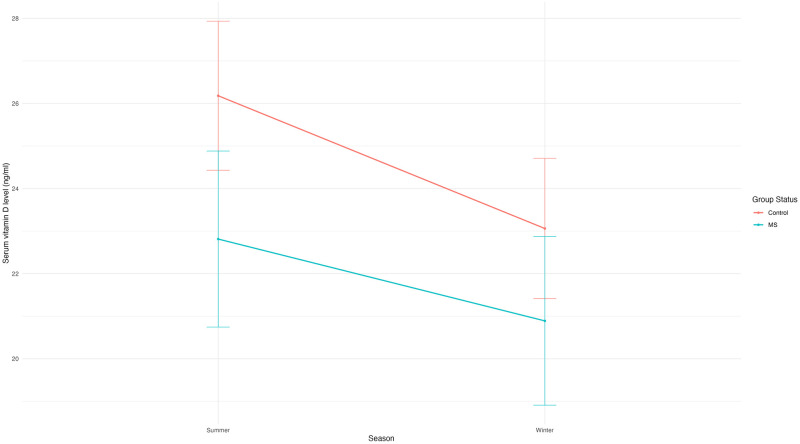
Serum vitamin D levels in persons with MS (cases) and persons without MS (controls). Changes in mean serum vitamin D levels (ng/ml) between summer and winter season stratified by group status (persons with MS, controls).

Median (interquartile range) serum vitamin D levels were lower in cases than controls, both in summer [23.00 ng/ml (11.30–31.50) vs. 25.0 ng/ml (19.25–32.0)] and in winter [21.0 ng/ml (10.6–27.6) vs. 23.5 ng/ml (15.55–28.60)] (Fig 1). According to IOF and ESE criteria, serum vitamin D deficiency was more prevalent in the MS group in both seasons compared with controls (summer: 41.9% (cases) vs. 32.3% (controls); winter: 48.4% (cases) vs. 38.7% (controls)). However, serum vitamin D insufficiency was less common in the MS group than in the control group (summer, 22.6% (cases) vs. 32.3% (controls); winter, 32.3% (cases) vs. 41.9% (controls)). Both groups experienced a decrease in mean (SD) serum vitamin D levels from summer to winter. However, the reduction was smaller in the MS group [1.92 ng/ml (2.34)] compared with the control group [3.12 ng/ml (3.28)].

### Factors associated with serum vitamin D levels

**Fig 2:**
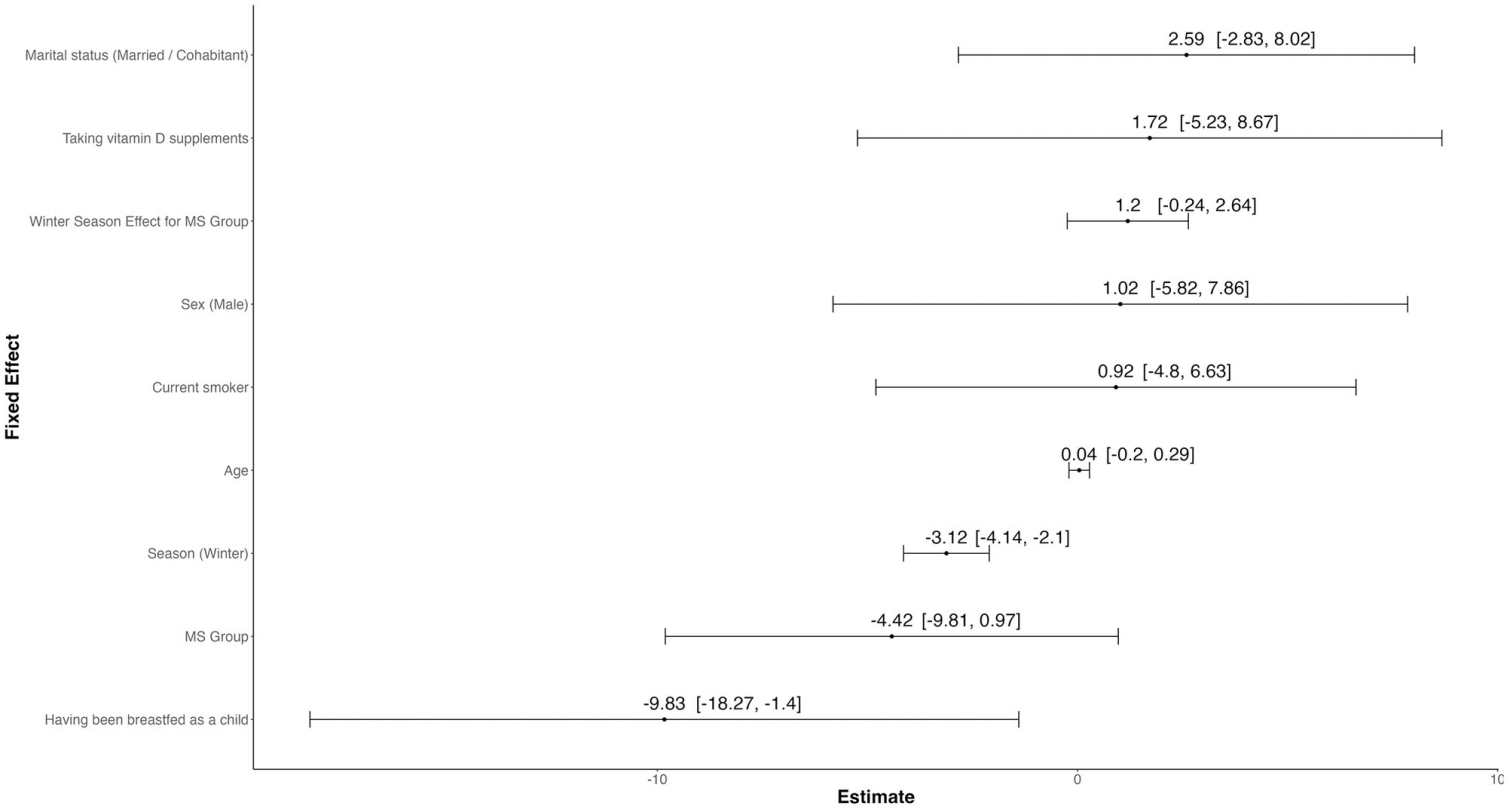
Results from the mixed-effects regression model. Estimates of fixed effects from linear mixed-effects regression showing the estimated associations of various factors on serum vitamin D levels. Point estimates and 95% confidence intervals are displayed for each factor.

Fig 2 illustrates point estimates and corresponding 95% confidence intervals from the multivariable linear mixed-effects regression model (detailed model output is shown in S1 Table). The regression analysis revealed a non-significant reduction of 4.42 ng/ml in serum vitamin D levels among MS patients relative to the control group, independent of season. In the control group, a significant seasonal effect was observed for serum vitamin D levels, with a marked decrease of 3.12 ng/ml during the winter season compared to the summer season (*p* < 0.001). Although the interaction between season and group status was not statistically significant, the analysis indicated a less pronounced seasonal decline in serum vitamin D levels among MS patients, with an estimated decrease of 1.92 ng/ml in the winter compared to the summer. This represents a 1.20 ng/ml smaller decrease compared to the non-MS group.

A history of having been breastfed as a child was significantly associated with lower serum vitamin D levels, showing a reduction of 9.83 ng/ml (*p* < 0.05). Marital status (married or cohabitant vs. single/divorced/widowed), vitamin D supplementation (yes vs. no), sex (male vs. female), and smoking status (current smoker vs. current non-smoker) were positively associated with serum vitamin D levels, yet these associations did not achieve statistical significance. The generalized mixed-effects analysis showed similar results (S2 Table), with effects generally trending in the same direction but being less pronounced. Also, the sensitivity analysis excluding persons taking vitamin D supplements yielded comparable results (S3 Table).

## Discussion

Vitamin D has been identified as a contributing factor to MS onset and progression. Therefore, a deeper understanding of environmental, behavioural, or nutritional aspects that influence vitamin D levels is important to derive context- and culture-specific health recommendations. Our study compared vitamin D levels in Mongolia between persons with MS and controls without the disease to examine differences, seasonal changes, as well as behavioural or nutritional factors that influence vitamin D. After controlling for potential confounding factors, we found that persons with MS had a tendency for lower vitamin D levels, both in summer and in winter, as well as a greater likelihood for a vitamin D deficiency. But, neither of these changes reached statistical significance. Having been breastfed as a child was the only factor found to be associated with lower vitamin D in a multivariable analysis. However, the robustness and validity of this finding need further confirmation.

Overall, the data suggest that persons with MS have lower vitamin D levels and point to a seasonal variation, but the statistical evidence for an effect is limited. Nevertheless, our observations fall in line with a host of other studies [21].

We can only speculate about the cause for lower vitamin D levels among persons with MS in our sample. The much lower percentage of persons reporting to spend at least 30 minutes daily in sunlight (22% of persons with MS compared to 100% in controls) suggests a behavioural component. Spending less time outside could also be related to impaired mobility, which was quite frequent in our sample of persons with MS (almost 30% of the MS patients reported being reliant on a wheelchair). Furthermore, some studies recommend vitamin D supplementation in persons with MS [22, 23].

Indeed, only 6 of 31 persons with MS reported taking vitamin D supplementation. Yet, the existing literature is unclear whether vitamin D supplementation should be more widely recommended to modify the disease course in persons with MS. Current evidence, including studies from randomized controlled trials [24], does not suggest a benefit on relapse rate or disease progression in persons with MS. Vitamin D supplementation may also be recommendable for other reasons, such as for the prevention of osteoporosis.

This study has strengths and limitations. Overall, the study is among the first to describe MS management and the situation of persons with MS in Mongolia, a country with constrained resources and means to treat MS. Nevertheless, our study sample is limited, and the generalizability for the larger population of persons with MS in Mongolia needs further investigation. Post-hoc power analyses indicated that our study may have been underpowered to detect certain effects. Specifically, to achieve 80% power for detecting the main effect of disease status (MS vs. control), a sample size of 93 participants per group would have been required. However, the actual power was only 38.0%. Similarly, detecting the interaction effect between season and disease status with 80% power would have required a sample size of 98 participants per group, whereas the actual power was 37.8%.

Furthermore, controls were recruited in a hospital setting and are therefore possibly not representative of the general population, for example, due to a higher likelihood for chronic illnesses [25]. Additionally, the lack of detailed information on alcohol and tobacco use, such as frequency, quantity, and duration, is a limitation that may have affected our ability to detect potential associations between these behaviors and serum vitamin D levels. Therefore, future studies should consider collecting more comprehensive information about these behaviors.

Moreover, the exclusion of participants using vitamin D and calcium supplements was not applied as specified in the study protocol, potentially influencing the study’s vitamin D-related findings. However, since the number of cases and controls taking vitamin D was balanced and we accounted for this variable in our regression models, we believe that our conclusions remain consistent. To further address this limitation, we conducted a sensitivity analysis excluding individuals taking vitamin D supplements. The results were consistent with our main findings, reinforcing the consistency of our conclusions.

Lastly, future studies should also consider exploring the effect of baseline vitamin D levels on future MS activity, progression, or relapse rate in the Mongolian population.

To conclude, our study seems to confirm that vitamin D deficiency is widespread among persons with MS. The relevance of this finding extends beyond MS management and may also be relevant, for example, for osteoporosis prevention. Furthermore, our study noted relevant behavioural differences between persons with MS and controls, which may help to refine MS-specific recommendation messages, for example, concerning nutrition, sun exposure, and vitamin D supplementation.

## Supporting information

S1 Fig Results from the mixed-effects regression model for individuals not supplementing vitamin D. Estimates of fixed effects from linear mixed-effects regression model, showing the estimated associations of various factors on serum vitamin D levels. Point estimates and 95% confidence intervals are displayed for each factor.
(DOCX)

S1 Table Results from the linear mixed-effects regression model. (DOCX)

S2 Table Results from the generalized mixed-effects regression model. (DOCX)

S3 Table Results from the linear mixed-effects regression model for the sample not supplementing vitamin D. (DOCX)
