# Supplementary material for: Factors associated with vitamin D levels in Mongolian patients with multiple sclerosis
Source: PLoS One. 2025 Jan 24;20(1):e0317279. doi: 10.1371/journal.pone.0317279 (PMC11760029; doi:10.1371/journal.pone.0317279)
Supplement: S1 Fig — Estimates of fixed effects from linear mixed-effects regression model, showing the estimated associations of various factors on serum vitamin D levels. Point estimates and 95% confidence intervals are displayed for each factor. (DOCX) [file pone.0317279.s001.docx]

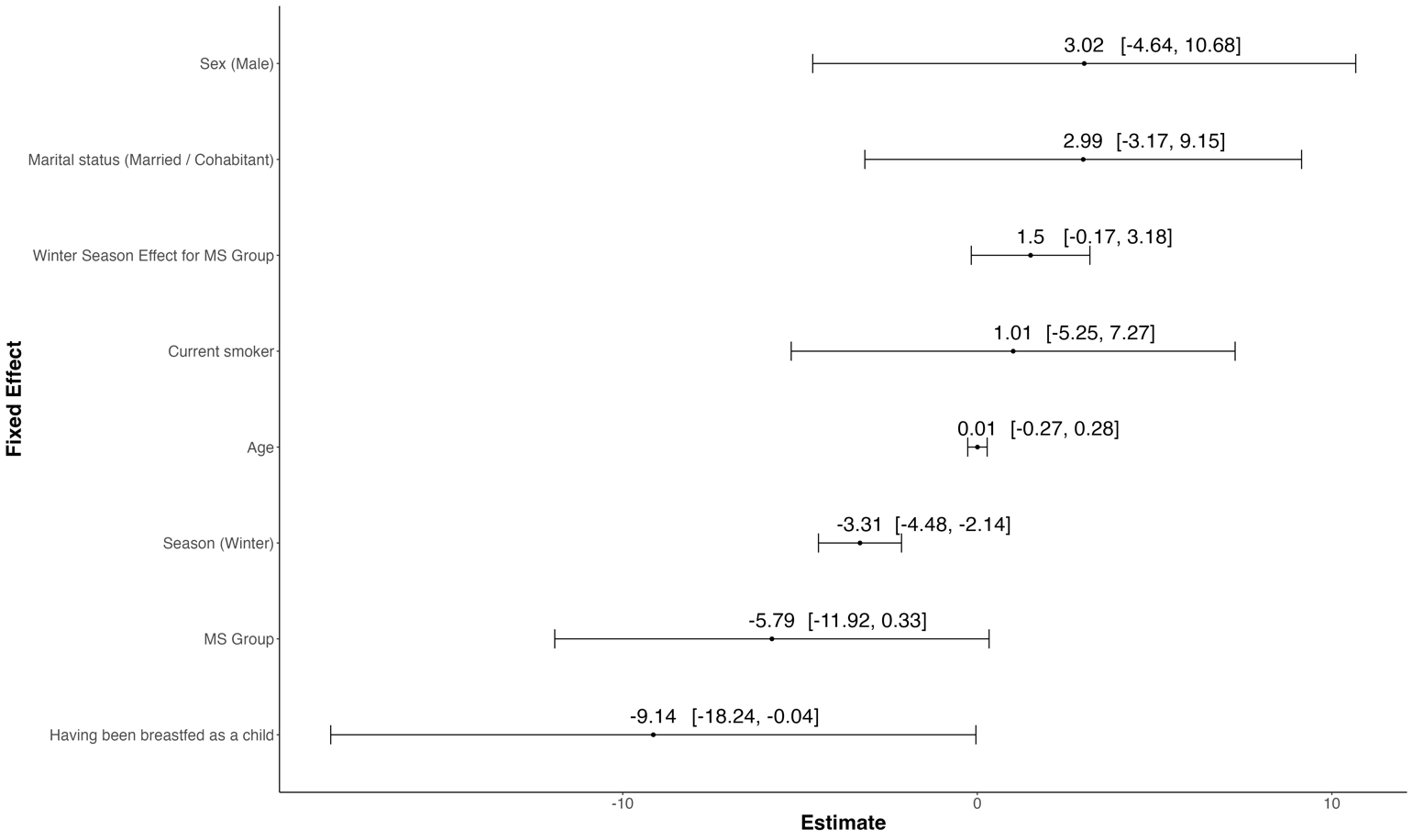


**S1 Fig**. **Results from the mixed-effects regression model for individuals not supplementing vitamin D.** Estimates of fixed effects from linear mixed-effects regression model, showing the estimated associations of various factors on serum vitamin D levels. Point estimates and 95% confidence intervals are displayed for each factor.
