# Supplementary material for: Factors associated with vitamin D levels in Mongolian patients with multiple sclerosis
Source: PLoS One. 2025 Jan 24;20(1):e0317279. doi: 10.1371/journal.pone.0317279 (PMC11760029; doi:10.1371/journal.pone.0317279)
Supplement: S1 Table — (DOCX) [file pone.0317279.s002.docx]

|  | **Vitamin D level** | | |
| --- | --- | --- | --- |
| Predictors | Estimates | 95% Confidence Interval | *p* |
| **Fixed Effects** | | | |
| Winter season [Ref = summer season] | -3.12 | -4.15 – -2.09 | <0.001 |
| MS group [Ref = control group] | -4.42 | -9.87 – 1.03 | 0.111 |
| Sex male [Ref = female] | 1.02 | -5.89 – 7.93 | 0.771 |
| Age | 0.04 | -0.21 – 0.29 | 0.739 |
| Currently smoking  [Ref = non-smoker] | 0.92 | -4.86 – 6.69 | 0.754 |
| Taking vitamin D supplement  [Ref = no vitamin D supplements] | 1.72 | -5.31 – 8.75 | 0.629 |
| Marital status; married / cohabitant  [Ref = single/divorced/widowed] | 2.59 | -2.89 – 8.08 | 0.351 |
| Having been breastfed as a child  [Ref = not been breastfed as a child] | -9.83 | -18.36 – -1.30 | 0.024 |
| Winter season [Ref = summer season] × MS group [Ref = control group] | 1.20 | -0.26 – 2.65 | 0.107 |
| **Random Effects** | | | |
| σ^2^ | 4.19 | | |
| τ_00_ _ID_ | 104.33 | | |
| ICC | 0.96 | | |
| N_ID_ | 62 | | |
| Observations | 124 | | |
| Marginal R^2^ / Conditional R^2^ | 0.113 / 0.966 | | |

**S1 Table**. **Results from the linear mixed-effects regression model.**
