# Supplementary material for: Factors associated with vitamin D levels in Mongolian patients with multiple sclerosis
Source: PLoS One. 2025 Jan 24;20(1):e0317279. doi: 10.1371/journal.pone.0317279 (PMC11760029; doi:10.1371/journal.pone.0317279)
Supplement: S2 Table — (DOCX) [file pone.0317279.s003.docx]

|  | **Vitamin D level** | | |
| --- | --- | --- | --- |
| Predictors | Estimates | 95% Confidence Interval | *p* |
| **Fixed Effects** | | | |
| Winter season [Ref = summer season] | -2.14 | -4.19 – -0.09 | 0.041 |
| MS group [Ref = control group] | -0.28 | -2.83 – 2.27 | 0.832 |
| Sex male [Ref = female] | 0.31 | -2.65 – 3.28 | 0.836 |
| Age | -0.02 | -0.13 – 0.09 | 0.667 |
| Currently smoking  [Ref = non-smoker] | 1.22 | -1.29 – 3.73 | 0.339 |
| Taking vitamin D supplement  [Ref = no vitamin D supplements] | 0.76 | -2.28 – 3.79 | 0.625 |
| Marital status; married / cohabitant  [Ref = single/divorced/widowed] | 1.47 | -0.97 – 3.91 | 0.237 |
| Having been breastfed as a child  [Ref = not been breastfed as a child] | -2.84 | -6.36 – 0.69 | 0.115 |
| Winter season [Ref = summer season] × MS group [Ref = control group] | 0.16 | -2.66 – 2.98 | 0.913 |
| **Random Effects** | | | |
| σ^2^ | 3.29 | | |
| τ_00_ _ID_ | 12.22 | | |
| ICC | 0.79 | | |
| N_ID_ | 62 | | |
| Observations | 124 | | |
| Marginal R^2^ / Conditional R^2^ | 0.150 / 0.820 | | |

**S2 Table.** **Results from the generalized mixed-effects regression model.**
