# Supplementary material for: Factors associated with vitamin D levels in Mongolian patients with multiple sclerosis
Source: PLoS One. 2025 Jan 24;20(1):e0317279. doi: 10.1371/journal.pone.0317279 (PMC11760029; doi:10.1371/journal.pone.0317279)
Supplement: S3 Table — (DOCX) [file pone.0317279.s004.docx]

|  | **Vitamin D level** | | |
| --- | --- | --- | --- |
| Predictors | Estimates | 95% Confidence Interval | *p* |
| **Fixed Effects** | | | |
| Winter season [Ref = summer season] | -3.31 | -4.48 – -2.14 | <0.001 |
| MS group [Ref = control group] | -5.79 | -11.92 – 0.33 | 0.064 |
| Sex male [Ref = female] | 3.02 | -4.64 – 10.68 | 0.440 |
| Age | 0.01 | -0.27 – 0.28 | 0.971 |
| Currently smoking  [Ref = non-smoker] | 1.01 | -5.25 – 7.27 | 0.751 |
| Marital status; married / cohabitant  [Ref = single/divorced/widowed] | 2.99 | -3.17 – 9.15 | 0.342 |
| Having been breastfed as a child  [Ref = not been breastfed as a child] | -9.14 | -18.24 – -0.04 | 0.049 |
| Winter season [Ref = summer season] × MS group [Ref = control group] | 1.50 | -0.17 – 3.18 | 0.078 |
| **Random Effects** | | | |
| σ^2^ | 4.64 | | |
| τ_00_ _ID_ | 111.93 | | |
| ICC | 0.96 | | |
| N_ID_ | 51 | | |
| Observations | 102 | | |
| Marginal R^2^ / Conditional R^2^ | 0.132 / 0.965 | | |

**S3 Table.** **Results from the linear mixed-effects regression model for the sample not supplementing vitamin D.**
